# A comparison of *in vivo* MRI based cortical myelin mapping using T1w/T2w and R1 mapping at 3T

**DOI:** 10.1371/journal.pone.0218089

**Published:** 2019-07-03

**Authors:** Zahra Shams, David G. Norris, José P. Marques

**Affiliations:** Donders Centre for Cognitive Neuroimaging, Radboud University, Nijmegen, Netherlands; Linköping University, SWEDEN

## Abstract

In this manuscript, we compare two commonly used methods to perform cortical mapping based on myelination of the human neocortex. T1w/T2w and R1 maps with matched total acquisition times were obtained from a young cohort in randomized order and using a test–retest design. Both methodologies showed cortical myelin maps that enhanced similar anatomical features, namely primary sensory regions known to be myelin rich. T1w/T2w maps showed increased robustness to movement artifacts in comparison to R1 maps, while the test re-test reproducibility of both methods was comparable. Based on Brodmann parcellation, both methods showed comparable variability within each region. Having parcellated cortical myelin maps into VDG11b areas of 4a, 4p, 3a, 3b, 1, 2, V2, and MT, both methods behave identically with R1 showing an increased variability between subjects. In combination with the test re-test evaluation, we concluded that this increased variability between subjects reflects relevant tissue variability. A high level of correlation was found between the R1 and T1w/T2w regions with regions of higher deviations being co-localized with those where the transmit RF field deviated most from its nominal value. We conclude that R1 mapping strategies might be preferable when studying different population cohorts where cortical properties are expected to be altered while T1w/T2w mapping will have advantages when performing cortical based segmentation.

## Introduction

The identification of the spatial organization of the myelinated fibres throughout the cortex was the basis of myeloarchitectonic studies of the cerebral cortex, which became of interest in the early 20^th^ century [1]. Recently, various *in vivo* MRI-based methods have been developed for studying the cerebral cortex on the basis of its “myelin” content to help achieve the goal of identifying regions on the basis of their myeloarchitecture which has been suggested to be predictive of brain connectivity [2]. These have used three general approaches for cortical myelin mapping in MRI: longitudinal relaxation rate, R1 (inverse of the longitudinal relaxation time, T1) [3–8]; apparent transverse relaxation rate R2* (inverse of the apparent transverse relaxation time, T2*) [9,10]; T1w/ T2*w or T1w/T2w mapping [11,12]. The motivation to use such metrics, particularly longitudinal relaxation rate maps to study myelin distribution, originates from various ex-vivo studies that have shown a direct relationship between R1 and myelin content [13–15]. Particularly in the cortex, R_1_ variations reflect water mobility [16,17], even if the impact of iron content on R_1_ tissue contrast should not be fully neglected [18]. Exploring the high SNR offered by high field imaging, Geyer et al. (2011) obtained high resolution ex-vivo (0.6 mm isotropic) quantitative T1 maps, using the MP2RAGE sequence [19], to delineate the border between Brodmann area 3a and area 4. Its location was in good agreement with their observations from high-resolution post-mortem histological studies [5] of the same tissue section. The same group extended this work to ex-vivo samples of primary visual cortex and used proton induced X-ray imaging to quantify iron and myelin. A dependence of the measured longitudinal relaxation rate on the local concentrations of both myelin and iron was observed [8]. Although the term “myelin mapping” is commonly used in literature, there is growing evidence that the high sensitivity of these methods to myelin does not make them very specific. Other methods, including Myelin Water Fraction Imaging using multi-compartment T2 fitting approaches [20] and Magnetisation Transfer [21] included in multi-compartment relaxometry [22] could be more specific to myelin concentration. Yet, this higher specificity comes at the cost of a decrease in sensitivity which does not allow whole brain acquisitions at the resolution needed to study cortical myelin distributions. Furthermore, it has been shown that the correlation between myelin water fraction and T1w/T2w imaging is low across subcortical white matter suggesting these two methodologies could be sensitive to different microstructural properties [23]. Due to these nuances, in this manuscript the maps obtained will be referred to as cortical- rather than myelin maps.

Quantitative relaxometry measurements, such as R1 and R2* maps are reproducible either within individuals or across subjects, being directly comparable across different scanners without the need for extra intensity normalizations. In 2004, Fischl et al. presented a set of techniques for spatially mapping MR properties of brain gray matter, such as T1, across the cortical surface at 1.5 T. The generated maps of T1 over the cortical surface (acquired at 1.3 × 1 × 1 mm^3^) revealed shorter T1 values in some primary cortical regions [4]. Also at 1.5T Sigalovsky et al. (2006) showed in a study focused in the auditory cortex that surface maps of R1 (acquired at 1.3 × 1.0 × 1.3 mm^3^) were related to myelin content and varied spatially on the superior temporal lobe, allowing quantitative comparisons over various intra-lobe sub-regions of interest [17]. More recently, at 3T, using R1 mapping (acquired at 0.8 × 0.8 × 0.81 mm^3^) based on a variable flip angle method, Sereno et al. (2012) investigated the congruence of cortical surface-based fMRI retinotopy with quantitative R1 mapping at laminar level to localize visual areas based on myelination degree. The same group subsequently combined R1 mapping with functional tonotopic fMRI to define the auditory cortex, showing consistent spatial extent across scans and subjects on both R1 measures and functional mapping [3]. At 7T the MP2RAGE sequence has been more commonly used owing to its low sensitivity to B1 inhomogeneity and the possibility to obtain high resolution R1 maps. The latter have achieved 0.6–0.7 mm isotropic resolutions in ~11mins [5,24–26], while higher resolution protocols at 0.5mm isotropic resolution were acquired in ~30 mins [27]. These studies have allowed not only to study cortical variations across the whole brain, but also to develop new methods to create layers across the cortical depth and to study the different type of layer profiles. It should be noted that the single pool model, used in the MP2RAGE sequence, with one single relaxation time is a simplistic approximation. Bi-exponential behaviour of signal recovery has been shown at high field and in white matter regions [28] and is likely to be present at 3T in grey matter to a smaller degree.

When looking at the apparent transverse relaxation times across the neocortex, Cohen-Adad et al. investigated their correlation to myelin concentration and potential application in multiple sclerosis, using a surface-based analysis [9,29]. In addition to observing significant variations in the relaxation times across different Brodmann areas, it was also observed that T_2_* values had a non-negligible dependence on the orientation of the cortical surface with respect to B_0_, which could be attributed to the myelinated axons running through the cortex. See [30] for a comprehensive review on T_2_* cortical mapping, from its theory to its mapping in the cortex.

As an alternative to the type of quantitative imaging advocated in the previous paragraph, Glasser and Van Essen proposed a method for in-vivo myelin sensitive cortical parcellation by taking the ratio of T1w and T2w images [12]. Taking the ratio improves the contrast for myelin which is inverted between the two initial images, and removes receive field inhomogeneities while, attenuating residual signal intensity bias related to the transmit coil [12]. Areal borders were computed by calculating the gradient magnitude of the surface maps, which allowed the characterization of cortical areas based on relative myelin content. T1w/T2w cortical mapping was further developed during the Human Connectome Project to a higher resolution (0.7 mm isotropic) which results in higher quality cortical maps [31]. However, unlike R1 map that reflects the intrinsic NMR tissue property, the T1w/T2w method is a relative measure, whose contrast depends not only on effects such as transmit field biases, that cannot be fully removed from the ratio image at higher field strength [12,32], but also on the exact sequence settings, making it challenging to compare across different scanners where equivalent protocols cannot be ensured. It should be added that both these measurements are field dependent, as R1 and T2 values decrease with increasing magnetic field.

The aim of this study is to investigate at 3T the relationship between these two cortical mapping methods (quantitative R1 and T1w/T2w ratio) and evaluate how correlated the information from these surrogate maps of myelin is. Moreover, the robustness of each technique was assessed by evaluating the reproducibility and consistency of the cortical maps obtained across subjects and from separate runs on the same subject. The bias field independency of each method was studied by looking into the correlation between the maps of the left and right hemispheres. The SNR at which the cortical maps are obtained and its reproducibility was also evaluated.

## Methods

### Experimental protocols

All scans were performed on a 3T Siemens Magnetom Prisma system (Siemens, Erlangen, Germany) equipped with a 32-channel receive coil. Seventeen healthy subjects (24.7 ± 2.8) participated in this study after having given their informed consent. The study was covered by our blanket ethics CMO 2014/288 –“Image Human Cognition”, approved by the CMO Arnhem-Nijmegen.

All the following experiments were run twice for each subject, test and retest, with the subject being repositioned in the scanner in between the two sessions. The subjects’ heads were either simply held with head cushions or additionally fixed using a chin-rest (to avoid head translation and rotation in the head to foot direction) in the test and retest condition (the fixation was randomized). Also, in both the test and retest condition, the order of the T1w/T2w imaging protocols and R1 mapping protocols were randomized. Data are available from the Donders Institute for Brain, Cognition and Behaviour repository at http://hdl.handle.net/11633/di.dccn.DSC_3015046.03_479

#### Quantitative R1 maps acquisitions

The quantitative R_1_ maps were acquired using the MP2RAGE [19] sequence. Although the sequence has a reduced sensitivity to RF inhomogeneities, some residual bias still exists in the R1 maps and it increases with increasing numbers of excitations per MP2RAGE repetition time, as is necessary when aiming at high spatial resolution [6]. To overcome this problem the B_1_^+^ field was measured separately using a fast turboFLASH B1 mapping [33] sequence. The two resulting datasets were used to compute high resolution, full brain and bias field free R_1_ maps [6].

MP2RAGE sequence parameters were: TR/TI_1_/TI_2_ = 5.5/0.7/2.5 s, BW = 210 Hz/Px, Flip angles (α1/α2) = 6°/4°, parallel imaging = 3, TE/echo spacing = 3.28/7.5 (ms/ms), FOV = 256 × 256 × 204.8, matrix size = 320 × 320 × 256, Partial Fourier in slice encoding direction = 6/8 (resulting in 64 and 128 excitations before and after the k-space center), Tacq = 12min 30sec. To attain more accurate cortical surfaces, we acquired high spatial resolution images of 0.8 × 0.8 × 0.8 mm^3^.

The fast saturation recovery turbo FLASH acquisition had the following parameters: TR/TE = 10/2.23 (s/ms), BW = 490 Hz/Px, FA = 8°, spatial resolution = 3.3 × 3.3 × 2.5 mm^3^, Dist. Factor = 100%, Tacq = 20 sec.

#### T1w and T2w acquisitions

T1w and T2w scans were collected using a T1w magnetization-prepared rapid gradient echo (MPRAGE) and a T2w sampling perfection with application optimized contrast using different angle evolutions (SPACE) sequences adapted from the Human Connectome Project imaging protocol. The 3D MPRAGE images were acquired with the parameters of: TR = 2200 ms, TE = 2.64 ms, TI = 1100 ms, 11° flip angle, bandwidth = 170 Hz/pixel, echo spacing = 9.3 ms, FOV 256 mm × 320 mm × 179.2 mm, matrix 320 × 400 × 224, 0.8 mm isotropic resolution, iPAT = 3, and an acquisition time of 6 min and 2 sec. The acquired SPACE images had the following parameters: TR = 3200 ms, TE = 569 ms, variable flip angle optimized for T2 weighting, bandwidth = 579 Hz/pixel, echo spacing = 4.12 ms, Turbo Factor = 314, FOV 256 mm × 320 mm × 179.2 mm, matrix 320 × 400 × 224, 0.8 mm isotropic resolution, iPAT = 3 and acquisition time of 6 min and 19 sec. T1w and T2w images were receive bias field corrected as provided by the vendor based on additional reference volume coil scan (Siemen’s pre-scan normalize).

It should be noted that the parameters of R1 mapping and T1w/T2w ratio protocols were adjusted to ensure the same spatial resolution (0.8 mm isotropic resolution) and total acquisition time (~12min 30 sec) thus allowing SNR and efficiency comparisons between both protocols.

#### Field map acquisitions

B0 field maps were acquired for the purpose of correcting readout distortion in the T1w, T2w and R1 images using a dual-echo gradient echo sequence adapted from the Human Connectome Project imaging protocol (http://humanconnectome.org/study/hcp-young-adult/documentation/data-release/Q1_Release_Appendix_I.pdf) with delta TE = 2.46 ms. Other imaging parameters are as follows: TR = 731 ms, TE1 = 4.96 ms, 50° flip angle, bandwidth = 566 Hz/pixel, FOV = 104 mm × 90 mm × 72 mm, matrix = 208 × 180 × 144, 2 mm isotropic resolution, interleaved multi-slice mode and the acquisition time of 2 min and 15 sec.

### Data processing

T1w and T2w data were processed using the minimal HCP preprocessing pipeline [31] which was also applied to the R1 data with some modifications. The preprocessing pipeline includes PreFreeSurfer, FreeSurfer and PostFreesurfer pipelines with the goal of removing spatial distortions, reconstructing cortical surfaces and creating myelin maps in the CIFTI format which will be readable in the Connectome Workbench visualization software [34].

#### T1w/T2w ratio processing

The T1w/T2w ratio processing pipeline has been thoroughly explained in [31]. Therefore, we give a brief overview of the preprocessing steps as applied to our own data.

The PreFreeSurfer pipeline includes “acpc alignment”, initial brain extraction by using an MNI template brain mask to mask out each individual’s brain, readout distortion correction of both acquisitions, registration of undistorted T2w to the undistorted T1w image, bias field correction of T1w and T2w scans, and finally nonlinear registration to MNI space.

For readout distortion correction, a gradient echo field map with two magnitude images and a phase difference image was used. Then the fsl_prepare_fieldmap script was applied to generate the fieldmap. The resulting fieldmap was scaled according to the different readout dwell times of the T1w and T2w scans and registered to them before finally performing unwarping [31].

Despite the Siemens prescan normalization having been applied and the ratio also contributing to bias removal, residual bias field present was corrected in both T1w and T2w images, by using the square root of the product of the two images [31,35].

T1w×T2w≅(x×F)×(1x×F)=F (1)

In this phenomenological equation, x represents the T1w cortical contrast that is approximately the inverse of the cortical contrast in T2w image, and F is the bias field. The division of normalized bias field image by its smoothed version was thresholded to create a mask. Then the normalized bias field was extrapolated from mask region out to the whole FOV and smoothed with a 5 mm kernel. The resulting bias field was used to create corrected images from both the T1w and T2w images. The PreFreeSurfer pipeline was completed by warping the two sets of images to MNI template in order to have each subject’s scans in both native volume and MNI space.

The processing then proceeded to the FreeSurfer pipeline which uses the undistorted bias field corrected T1w image as input. The aim of this step is the reconstruction of white and pial cortical surfaces, and final registration to the surface template. In autorecon1 of FreeSurfer’s recon-all, the extracted brain from “PreFreeSurfer” pipeline was used to improve “mri_em_register” registration, generating a FreeSurfer brain mask. After “autorecon2” steps, the reconstructed white surfaces and all the volumes needed for FreeSurfer’s mris_make_surfaces were mapped to high resolution (0.8 mm) coordinates. Then the intensity normalization was performed on a high-resolution volume to correct the position of the white matter surfaces. Registration of T2w images to T1w images was tweaked using FreeSurfer’s boundary based registration which employed deformed white matter surfaces. FreeSurfer's folding-based surface registration was used for registration to fsaverage atlas. Another important step included in the HCP pipeline is the use of T2w images in addition to T1w to achieve more accurate pial surfaces, excluding any dura and blood vessels (see [31] for more detail). Subsequently, a PostFreeSurfer pipeline converted the FreeSurfer volumes and surfaces to NIFTI and GIFTI formats, creating a FreeSurfer ribbon file at full resolution. The same pipeline was used to generate surface cortical maps on individual subject's native-mesh and on the high resolution (164k_fs_LR) Conte69 registered standard mesh. While the approach in Eq 1 has been shown to provide robust “myelin maps” [12], for cortical segmentation an extra procedure was performed to remove the residual bias field from the individual maps [31]. In this bias correction step, the group average myelin map on the Conte69 mesh was used as a reference for T1w/T2w myelin map to estimate the residual bias field presented in each individual’s original myelin map and finally to correct it [31]. At the end, we had both the original and the bias corrected T1w/T2w maps. For a more complete overview of the pipeline visit Glasser et al.’s publication on the HCP processing pipeline [31].

#### R_1_ calculation

For T1w MP2RAGE data, we perform similar processing steps to those described in T1w/T2w ratio processing. Prior to them, the salt and pepper noise of the background was masked out from MP2RAGE images by thresholding the two original images. Then the resulting images were cropped in the slice direction with FSL’s robustfov tool (www.fmrib.ox.ac.uk/fsl, b = 180). The cropped volume was registered to MNI152 with flirt (12 DOF) using spline interpolation. After acpc alignment, the brain extraction was performed based on 12 DOF linear flirt registration to the 2mm reference image by spline interpolation, followed by nonlinear fnirt registration to the low resolution (2mm) T1w MNI template. The output image was overwritten with a spline interpolated high resolution version. Finally, the template brain mask was used to mask the acpc aligned input image by inverted warp. The brain extraction step was followed by readout distortion correction. We followed the same procedure as for T1w/T2w ratio distortion correction—warping the fieldmap generated from the phase difference of gradient echo acquisition to the T1w image and creating a warpfield that corrected distortions in the readout direction (associated with a dwell time of 0.152 ms). To correct the B1 transmit inhomogeneity in T1w MP2RAGE image, first the low resolution magnitude image from a turbo fast low-angle-shot (TurboFLASH) readout pulse sequence with centric k-space reordering was co-registered to the high resolution MP2RAGE image with proton density contrast using FSL’s flirt. Then the transform matrix was applied to the B1^+^ map to obtain the B1^+^ map co-registered to the MP2RAGE image. The co-registered B1 map was masked using the brain extracted MP2RAGE image. The masked image was divided by its smoothed version which was done by within-mask smoothing of the co-registered masked B1 map with a Gaussian 5 mm kernel (the same degree of smoothing as for T1w/T2w bias field). A mask was then created using a threshold at Mean– 2*STD which was followed by island-removing. The B1 map processing was completed by extrapolating brain extracted image from mask region out to whole FOV https://github.com/Washington-University/Pipelines/blob/master/PreFreeSurfer/scripts/BiasFieldCorrection_sqrtT1wXT1w.sh). This output B1 map was the input to the MP2RAGE correction algorithm (https://github.com/JosePMarques/MP2RAGE-related-scripts), where B1value specific MP2RAGE intensity vs T1 lookup tables were used to compute the local T1 (1/R1) values [6]. The corrected R1 map was created by inverting the corrected MP2RAGE image (in the ms scale) and scaling that in mHz. Then it was registered to the T1w MPRAGE image using FreeSurfer’s boundary-based registration (which outperformed flirt with 6 degrees of freedom in terms of registration accuracy) to register the MP2RAGE gray matter white matter surfaces with the MPRAGE data. The purpose of this step was to ensure that any difference in the results was not due to the definition of the cortical surfaces because of contrast differences between them.

After bias-free R1calculation and registration to MPRAGE data, atlas registration to MNI152 was performed exactly in the same way as the T1w/T2w data with FSL’s flirt and fnirt tools.

#### Cortical maps

The visualisation of the cortical maps and the comparison across subjects used surface-based analysis. To create the surface cortical maps, the scripts in the HCP’s PostFreeSurfer pipeline were followed (with the steps mentioned in 3.2.1.) and applied both to R1 (with some modifications and disregarding the residual bias field correction step) and the T1w and T2w ratio images. To map the volumes on the surface, the data from R1 and T1w/T2w volumes of each individual were mapped onto the mid-thickness surface using the “myelin style” method (https://github.com/Washington-University/Pipelines/blob/master/PostFreeSurfer/scripts/CreateMyelinMaps.sh), except the applied Gaussian kernel for weighting voxels had the FWHM equalled to 1 (σ ≈ 0.425 mm). The contrast in WM of the two contrasts (R1 and T1w/T2w) is remarkably different, thus not integrating through the whole cortical depth avoids introducing confounds in the cortical comparison associated with partial volume be it at the WM GM or pial surfaces or originating from the through-layer myelin variation [7,26,36]. Then each individual’s cortical map was smoothed on the mid-thickness surface by a Gaussian kernel function with the FWHM of 4mm (*σ*≈1.698 mm) to reduce noise. Individual subject’s maps were also resampled to their corresponding 164k_fs_LR standard surface mesh which had previously been obtained by registering (with Freesurfer) each subject’s native-mesh surfaces to the Conte69 population-average ones with correspondence between the left and right hemispheres [31,37]. All the surfaces were regenerated for the retest session and taken to the same high resolution 164k_fs_LR atlas surface.

#### Cortical gradient

The gradient magnitude of the cortical maps, which is the first spatial derivative of the maps, was computed using hcp “wb_command”. First, "-cifti-create-dense-scalar” was used to create dense scalar files of the cortical maps from the metric files and then “-cifti-average” was used to generate the maps for the group average. The sigma for the Gaussian surface and volume smoothing kernels which were applied prior to the gradient computation were 2.35 mm and 0.1 mm respectively. Then, the gradient magnitude of the average cortical map was computed by using “–cifti-gradient”.

#### Group average spatial maps and atlases

The group average midthickness surface used in areal gradient computation was HCP S900 fs_LR midthickness surface [37,38], and the cortical parcellations chosen on this work were those defined by Brodmann (1909) based on cytoarchitecture [39] and VDG11b 52-surface-mapped cortical areas [37] as shown in S1 Fig and can be found in 32k_fs_LR mesh available in BALSA database [38]. To make the most of the high spatial resolution of the data, we resampled these atlases to 164k mesh surfaces.

### Data analysis

All the analyses were performed in MATLAB (version 2012b) where the R1 maps, original T1w/T2w cortical maps and their bias corrected version in 164k_fs_LR standard surface mesh were imported with the “gifti” tool, version 1.6 (http://www.artefact.tk/software/matlab/gifti). Additionally, the very inflated 164k_fs_LR standard surface mesh and high resolution Brodmann and VDG11 atlases obtained as described in the previous section were read in MATLAB to allow the visualization of the cortical maps and characterization of the cortical regions respectively. The left and right hemisphere of the cortical maps, the surface map and atlas label files were kept separated throughout the analysis.

To investigate how head fixation affects the created cortical maps and which of these two protocols is more sensitive to subject movement, we divided the data in two different groups with head fixation and without head fixation (in each of these two groups the test and retest are equally represented). Each of the subgroups contained all 17 subjects. Subsequently all data was visually inspected (ZS and JPM) to detect artefacts. This process reduced the initial sample of each group to 12 subjects where good quality scans were found on both cortical mapping protocols and in both the Test and Re-Test (Head Fixation vs. No Head Fixation) conditions. Table 1 shows the number of datasets where reconstruction artefacts (movement, fat rings or parallel imaging related) were found. The level of artefacts was generally low.

**Table 1:** Number of Artifactual datasets by each of the two T1w/T2w and R1 protocols which had been visually inspected.

Artifactual Datasets	T1w/T2w (T1w-MPRAGE and T2w-SPACE images)	R1 (MP2RAGE image)
**Head Fixation**	1/17	3/17
**No Head Fixation**	2/17	4/17

Evaluation of head fixation effects was performed on all 17 subjects (see Table 1). Further comparisons between R1 and T1w/T2w were carried out on 14 subjects with negligible reconstruction artefacts in both MP2RAGE and T1w/T2w head fixed scans (head fixed scans of both protocols).

#### Head fixation impact on cortical mapping

The following analyses were performed on each of the T1w/T2w ratio and R1 maps separately:

We labelled cortical areas based on the high resolution (164K) Brodmann atlas (resampled from the low resolution atlas available in the BALSA database) on the group average (cortical maps averaged across all 17 subjects). The average of all vertices values within each Brodmann area (BA) was subsequently calculated for the group average. Then linear regression was performed to the average values within each Brodmann regions between head fixation and non-fixation. Moreover, standard deviation within each cortical region for both head fixation and non-fixation maps on the group average was computed to study the effect of fixation in reducing the standard deviation within each cortical region. The other effect to be analyzed was the impact of fixation in variance across subjects. For this, the standard deviation for each BA was obtained across subjects and divided by the number of subjects. The same analysis was repeated for the subset of 12 artefact-free datasets.

#### Correlation between cortical mapping methods

To evaluate whether the two methods were measuring the same quantity (or highly correlated quantities) the degree of linearity between T1w/T2w (original and Bias corrected maps) and R1 maps was evaluated. We further tried to understand the origin of mismatch between the two cortical maps. To compare the variance within each Brodmann area for each protocol, standard deviation of the mapped values in each Brodmann region was measured on the group average. This metric could be interpreted as an artifactual noise in each region if it was naively assumed that within each BA the myelin map is constant. To determine the ability of the two methods in discriminating between neighboring areas, t-tests were applied to selected pairs of cortical regions in heavily myelinated areas of the occipital lobe and in the central sulcus of the group average (on BC maps of T1w/T2w). For the characterization of the neighbouring regions in visual and motor cortices, 52-area VDG11b parcellation was used on group average (see those parcellations in S1 Fig). The Measures of Effect Size (MES, Matlab toolbox) was used to compute the effect size and related quantities for our independent samples T-test [40]. The two group samples on which MES was performed consisted of the cortical mapping values in all the vertices of one given region versus the second region of interest. Because of the different sample sizes from each group, the effect size was weighted according to the relative size of each group sample by considering the Cohen’s d [40]. P-value, degree of freedom (DOF) and the value for Cohen’s d as the measure of effect size for some pairs of regions in sensory-motor and visual cortices are computed. Moreover, consistency and reproducibility of the maps across the experiments was evaluated by the variance over the 14 subjects for each method.

#### Evaluation of test-retest on T1w/T2w vs R1 maps

For the purpose of investigating the reproducibility of each of the two protocols, deviation maps were computed. The absolute value of the difference between the Test and Retest cortical maps was computed on each subject. The deviation maps of the two protocols were then normalised by the range of the cortical values found on the group. The normalized deviation maps were then averaged over the 12 subjects devoid of artefacts.

#### Left vs Right hemisphere reproducibility of T1w/T2w and R1

If it is assumed that there are no large cortical property differences between equivalent regions in the left and right hemispheres, any differences found in the cortical maps should be associated with B1 or B0 field inhomogeneities. This signal intensity bias dependency of each of the maps was investigated by looking at left vs right hemisphere correlation plots for each technique.

## Results

### General quality and reproducibility of cortical maps

**Fig 1:**
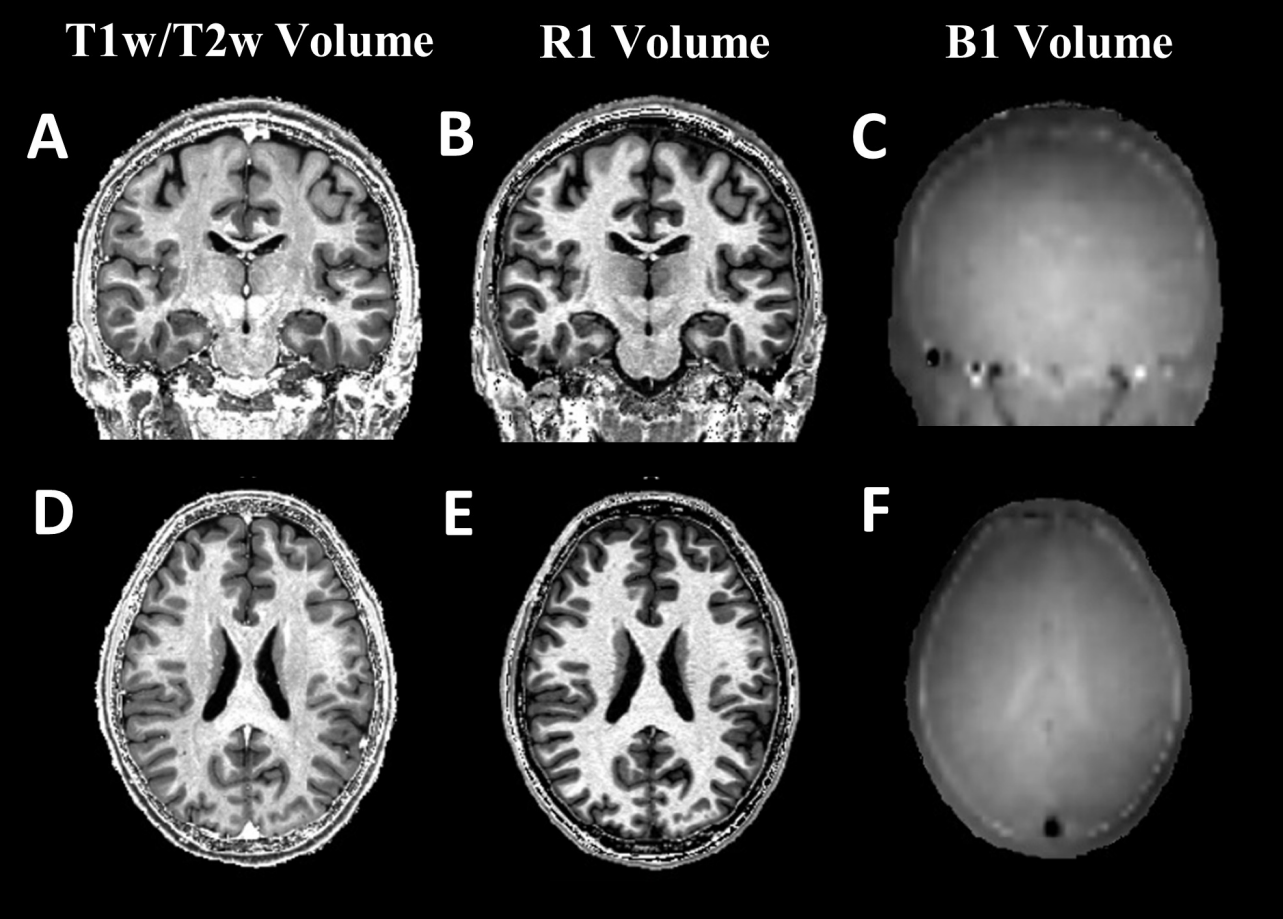
A single subject coronal (top panels) and axial (bottom panels) view of T1w/T2w (A, D), R1 (B, E) and B1+ (C, F). In C and F, slight left and right hemisphere asymmetry and large variation of the transmit field from the inner to the outer part brain can be observed.

In Fig 1, representative coronal (A-C) and transversal slices (D-F) of T1w/T2w, R1 and transmit bias field of a single subject are shown. While both T1w/T2w and R1 show strong CSF-GM-WM contrast, the contrast within white matter is notably different and, for example, T1w/T2w images have increased intensity in iron rich deep gray matter structures. Note that, while there is some left right asymmetry in the brain transmit B1 field, the biggest variation is from inner (having high values) to outer (having low B1 values) regions.

**Fig 2:**
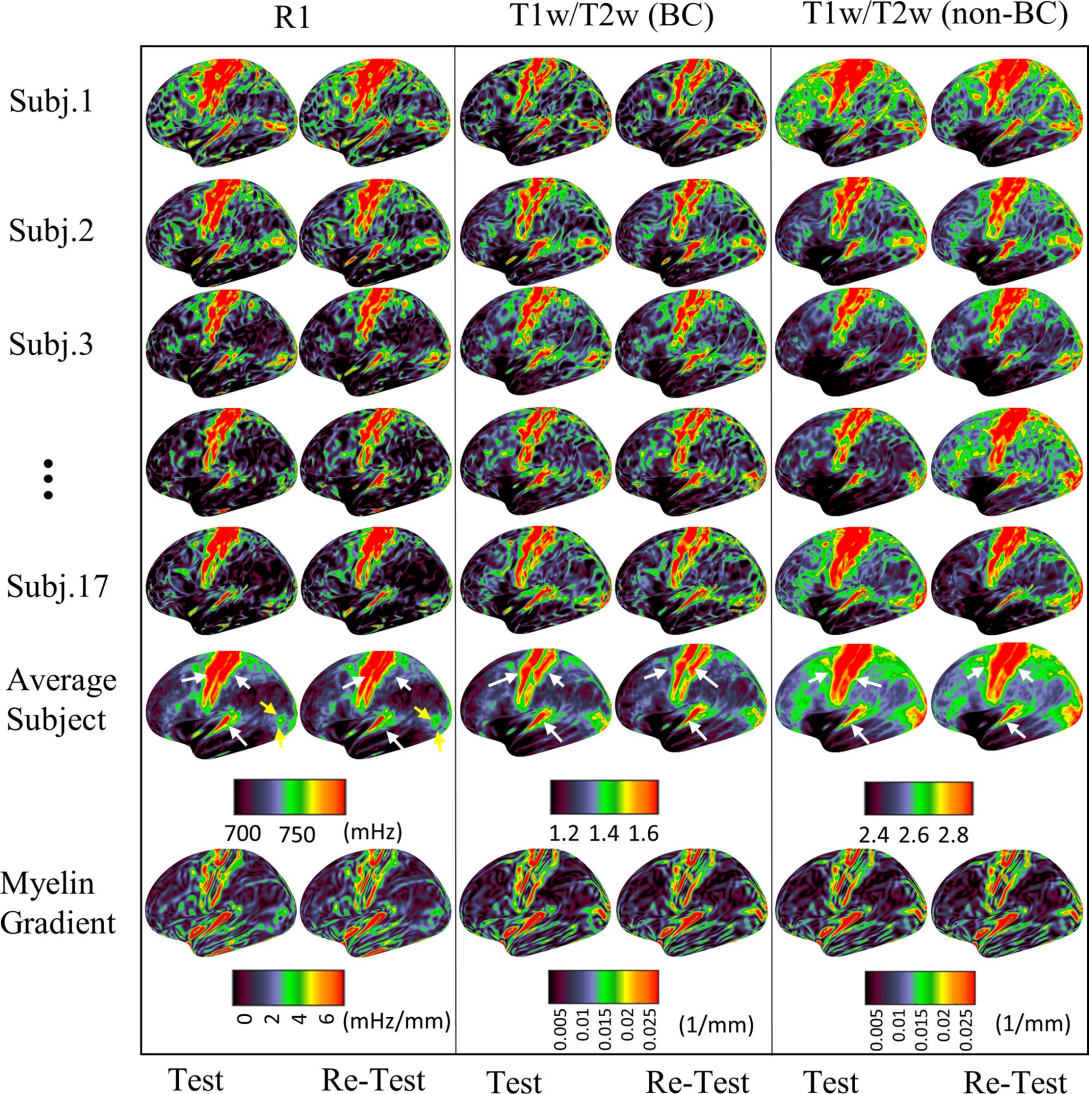
R1 and T1w/T2w Cortical maps in lateral view (Left hemisphere).Cortical maps of 5 individual subjects for left hemisphere represented on the high resolution fs_LR inflated surface. The two columns within each panel were obtained in the Test and Re-Test session. The middle and left panels are T1w/T2w maps with (BC) and without (non-BC) residual bias field correction. The last two rows show the group average maps and their gradients. Highly myelinated motor, sensory and auditory areas are highlighted with white arrows. The yellow arrows in R1 average maps indicate the middle temporal (MT) visual area as quite distinct from the BA18-V2. See S2 Fig for the medial view of the maps.

Cortical maps of the left hemisphere for 5 different subjects and the group average generated from the both T1w/T2w and R1 protocols in two sessions are shown in Fig 2 (lateral view) and S2 Fig (medial view). The middle and the right panels consist of the T1w/T2w with and without residual bias field correction (BC and non-BC respectively). This low frequency correction of T1w/T2w maps has a large effect on the individual subjects and the group average as can be observed by the reproducibility of the T1w/T2w BC and non-BC panels. Regions of heavy myelination (motor, sensory and auditory cortices pointed out with white arrows) and lightly myelinated areas are highly consistent between the two protocols. The same patterns can be observed in repeated experiments, showing high individual reproducibility of the surfaces for both R1 and T1w/T2w ratio protocols. Fig 2 demonstrates that cortical maps acquired in different sessions and with different protocols of the same subject share most cortical patterns, for example, note the shapes of the highlighted motor and sensory cortices, auditory, and MT+ regions (all highlighted with arrows) which are all clearly more reproducible within subject (and across techniques) than within each technique and across subjects. Despite this inter subject variability, the average maps still show the high myelin content in the expected cortical regions. Based on average maps, MT+ (pointed with light blue arrows on average R1 maps in Test and Retest) is more distinct in a way that it is a separated area from the neighbouring ones compared to T1w/T2w in which MT+ expands to V2 (see yellow arrows). The last row of the figure shows the surface gradient of the cortical maps, demonstrating the rapid changes in cortical myelination which helps delineation of the borders between cortical regions. The cortical maps for right hemisphere can be found in S3 Fig.

### Relevance of head fixation

Table 1 gives a first indication that the fixation system helped to reduce artifacts. Furthermore, it suggests that the R1 mapping strategy was more sensitive to subject movement than the T1w/T2w protocol. This was to be expected, although the total protocol has the same duration, the R1 mapping consists of one long acquisition, while the ratio approach consists of two shorter acquisitions (T1w-MPRAGE and T2w-SPACE acquisitions). Movement within each measurement is bound to be smaller for the shorter acquisitions and can be to some extent corrected in a post-processing step via co-registration.

**Fig 3:**
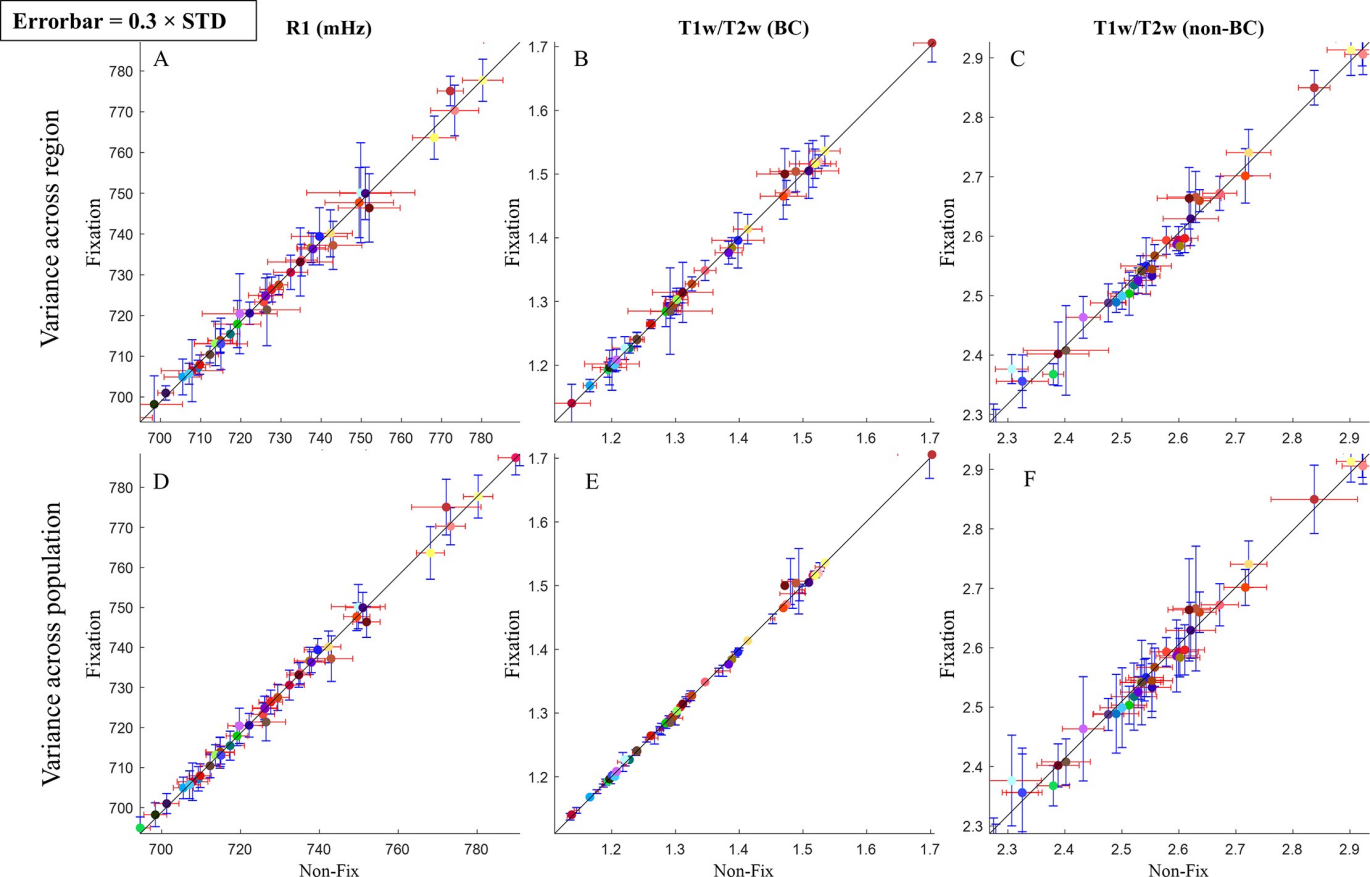
The relationship between test (Fixation) and retest (Non-Fix) in different cortical maps of R1 (A, D), residual bias field corrected T1w/T2w (B, E) and original T1w/T2w (C, F). Each point in the plots represents a particular Brodmann area with the value equals to the average value of the vertices within that region. The vertical and horizontal lines are the standard deviations in test and retest in each cortical region on the group average (A-C) and across the population (D-F). All the standard deviations in all of the plots are multiplied by 0.3 to facilitate visualization. Highly correlated test-retest values are depicted with fitted curves from linear regression.

Fig 3 shows plots of the cortical maps mean Brodmann are values in the Test (fixation) and Retest (non-fix) measurements for both techniques. It can be seen that a high degree of correlation between Test and Retest (Pearson’s R^2^>0.99) is present in both R1 map (Fig 3A and 3D) and T1w/T2w maps (Fig 3B, 3E, 3C and 3F). The errorbars in Fig 3A–3C represent the standard deviation within each region for the group average. Fig 3D–3F indicates standard error for each region value across subjects. Errorbars in plots Fig 3A–3C suggest similar deviations from the mean value within each region on group average for both Test and Re-Test conditions and for the different methodologies. As is also the case for the standard deviation of each area value across population only, when not correcting for the residual bias field in T1w/T2w (Fig 3D and 3E). Based on S4A–S4C Fig, in each cortical region, standard deviation has been hardly affected when we performed the same analysis except for low quality scan (12 artifact-free subjects) while the standard deviation over the population increases for R1 and bias corrected T1w/T2w maps, but comparable between Test and Retest (S4D and S4E Fig). These observations indicate that artifact-free images can better retain the natural myeloarchitectonic variations in each atlas-based BA, with artifacts essentially smoothing the maps. On the non-BC T1w/T2w data, variations across population increased when the artifactual data were included in the analysis, demonstrating its sensitivity to B1 changes in the presence of movement (compare Fig 3F and S4F Fig).

### Correlation between R1 and T1w/T2w maps

**Fig 4:**
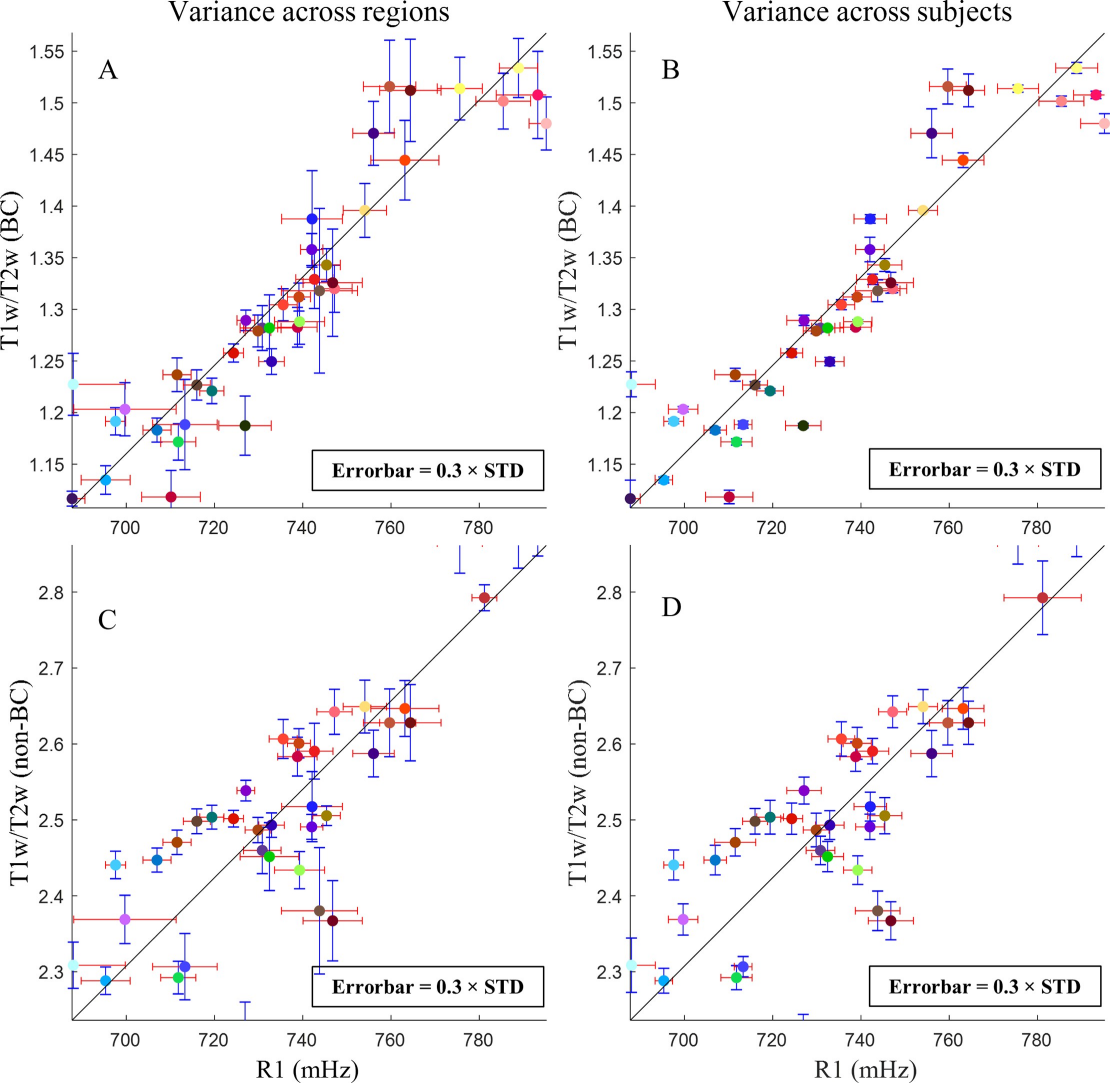
Correlation between R1 and T1w/T2w methods for characterizing cortical regions. Plots in (A, B) show the relationship between R1 map and bias corrected T1w/T2w, and in (C, D) is related to the comparison between R1 map and original T1w/T2w cortical map. The average of all vertices values in each Brodmann region is depicted in the colour corresponding to its label colour (see S1A Fig). the linear regression is shown in black curve. (A, C) Variance (multiplied by 0.3) in each BA on the group average and (B, D) across population are presented for both methods. Black arrows in (B) point to the regions with largest deviation from the fitted curve.

The relationships between R1 and T1w/T2w (BC and non-BC versions) maps are presented in Fig 4. A linear relationship (black curve) can be observed between R1 and bias corrected T1w/T2w ratio with Pearson’s R^2^ value of ~0.8 (Fig 4A and 4B), demonstrating that the two methods describe closely correlated features potentially related to cortical myelination. For assessing the correlation of the gradient maps, the linear regression results in R-square values of 0.62 and 0.60 for left and right hemisphere respectively which was done on all vertices of the average maps (BC version of the T1w/T2w map). Surprisingly, when considering only highest 25 percentile of the gradient values of the group average in both protocols, R-square values decreased to 0.33 and 0.27 for left and right hemispheres, showing that this correlation is not driven only by the largest gradients.

The degree of linearity between the two protocols decreased when not correcting for the residual B1^+^ in T1w/T2w map resulting in R^2^ ~0.74 (Fig 4C and 4D). At 3T this relationship can be characterized by the following equations.

$$\frac{T1w}{T2w}(\mathrm{BC})=0.0048\;R1-2.1870$$ (2)

$$\frac{T1w}{T2w}(\mathrm{non}‐\mathrm{BC})=0.0066\;R1-2.28$$ (3)

The Brodmann areas which show largest deviations from this linear trend are BA11 (orbitofrontal area), BA30 (in the isthmus of cingulate gyrus), BA27 (hippocampal area), BA32 (a subdivision of cingulate region) and BA37 (occipitotemporal area) which are indicated by black arrows in Fig 4B. Note that particularly the regions 27, 30, 32 and 37 are regions of relatively high transmit B1 field (see S5B Fig). This suggests that one of the methods was not able to address this specific bias correctly. Fig 4 has axis with different dimensionality and therefore the errorbars in the two axes cannot be directly comparable. To assist visual inspection, the regression curve calculated in Eqs 2 and 3 is shown, and the x and y axis ranges were chosen to render this trend close to an identity line, making the errorbars comparable. The standard deviation within each cortical region on the group average for each technique is shown in Fig 4A and 4C which is quite similar in most of the areas for both techniques. The mean variation between the subjects is smaller for bias corrected T1w/T2w method in a large number of areas (Fig 4B), indicating higher consistency of the obtained values across experiments as was already suggested by Fig 3B, and 3E, while the standard T1w/T2w maps have comparable variation cross subjects (Fig 4D). This suggests that the variance between subjects could be real and artificially removed during the bias correction process.

As observed in Fig 2, similar cortical patterns can be seen in different maps. However, a more careful inspection shows that the middle temporal visual area (MT/V5) is more distinguishable in the R1 average map while in the T1w/T2w the intensities are closer to those of V2 (visible in medial view). Moreover, there are subtle differences in motor-sensory cortical maps. The borders between the areas of 4, 3a and 3b is more visible in T1w/T2w maps. Table 2 indicates the results of a t-test on T1w/T2wBC and R1 maps for two neighboring regions of V2 and MT in visual cortex, and neighboring regions of 1, 2, 3a, 3b, 4a, 4p and 6 in motor cortex. According to the table, both methods have statistically significant P-value values less than 0.05. The effect size index of Cohen’s d has been calculated as well, in which Cohen’s d of < 05, = 0.5, > 0.5, > 1.2, > 2 represents small, medium, large, very large and huge effect sizes. For most of the entries, significant effect size can be observed for both T1w/T2w and R1 maps. As an example, t-test on MT and V2 results in a large effect size of 0.77 and 1.17 for T1w/T2w and R1 maps respectively, as well as very large effect sizes for 1–2, 4a-3a, 3b-4p and 4a-6 pairs. However, for 3a and 3b areas as well as regions of 1and 3b, R1 method results in smaller effect sizes of 0.47 and 0.24 comparing to those of T1w/T2w with the large values of 1.51 and 0.76 respectively.

**Table 2:** The result of t-test analysis performed on some neighboring regions of the central sulcus and occipital lobe in T1w/T2w and R1 group average.

		V2, MT	1, 2	3a, 3b	1, 3b	4a, 3a	3b, 4p	4a, 6
P-value	**T1w/T2w**	0	0	0	0	0	0	0
	**R1**							
DOF	**T1w/T2w**	4173	4257	3866	3604	4161	4052	10284
	**R1**							
Cohen’s d	**T1w/T2w**	0.77	2.18	1.51	0.76	2.28	1.21	2.99
	**R1**	1.17	2.51	0.47	0.24	1.63	1.20	2.94

DOF, degree of freedom.

Significant difference in Cohen’s d between the two methods has been underlined.

### Test-Retest evaluation

**Fig 5:**
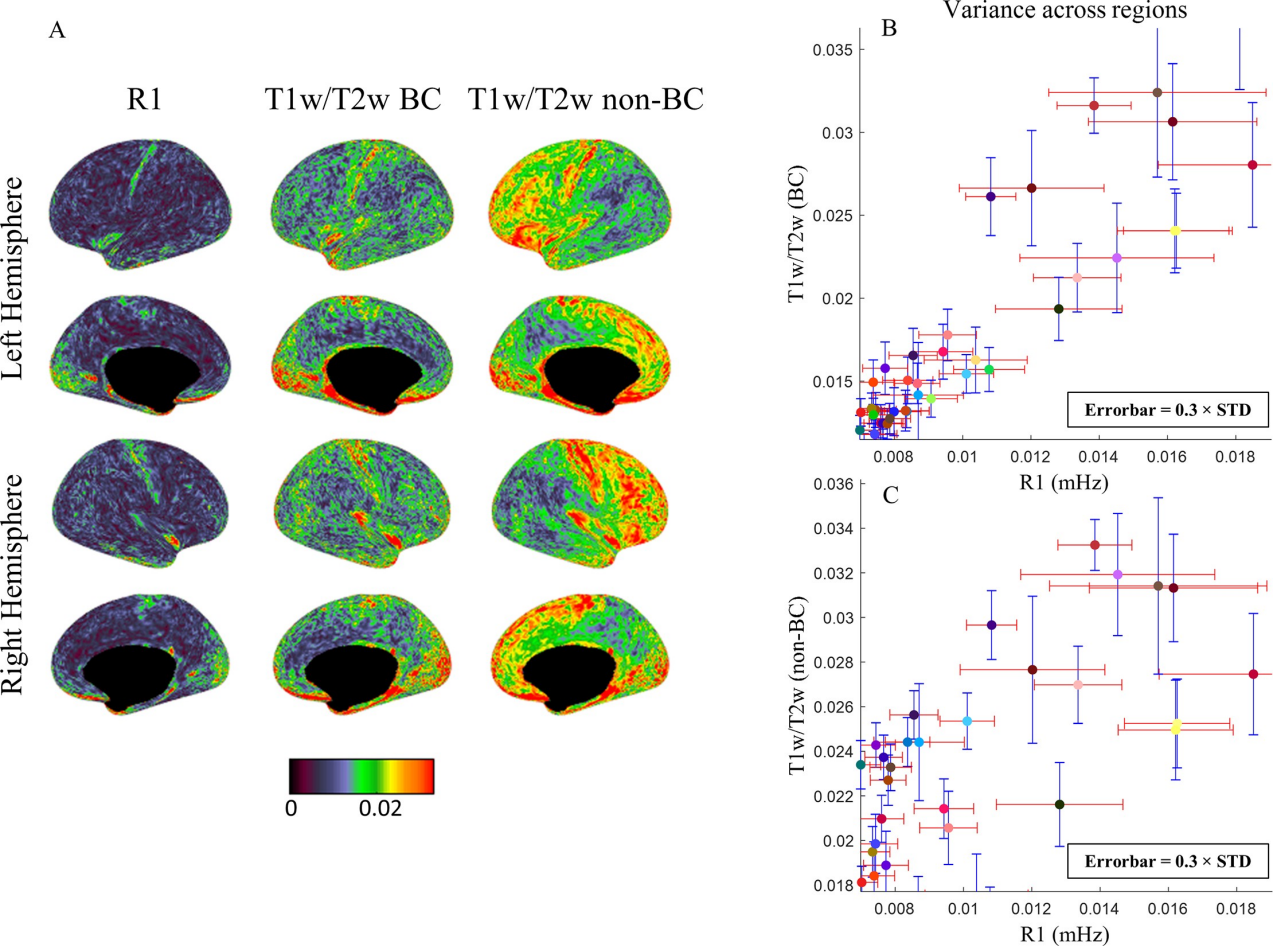
Average of Deviation maps across the subjects for both left and right hemispheres (12 artifact-free datasets). (A) surface representation of average deviation maps of R1, bias corrected T1w/T2w and original T1w/T2w. Particularly large residual bias field exists in T1w/T2w non-BC maps in frontal lobe and with decreased intensity in the posterior. The color scale (from 0 to 0.025) is normalized by the range of cortical values found in the brain and thus 0.02 can be interpreted as a test-retest variation of 2% of the range of values typically found in the respective cortical maps. (B) comparison between variance of each cortical area in bias corrected T1w/T2w and R1 deviation maps shown by errorbars. The errobars are the standard deviation in each region of the deviation maps (shown in A). (C) Variance of Each Brodmann region in R1 deviation map vs. non-BC T1w/T2w.

To evaluate the precision of repeated measurements on an individual subject, mean deviation maps (over the 12 subjects free of artifacts) were created (see “Data analysis” section) and are shown for left and right hemisphere in Fig 5A. Deviation maps are standardized maps, making them comparable across different methods. It can be seen that R1 measurements have a high reproducibility with the cortical map variations between test and re-test, with the largest percent variations being found in regions of high “myelination”. Deviation maps for bias corrected T1w/T2w (Fig 5A, middle panel) show less reproducibility, resulting in larger errorbars in Fig 5B. Those errorbars represent the deviation from the mean in each Brodmann region for the average deviation map. When T1w/T2w are not corrected (non-BC T1w/T2w, Fig 5A right panel), increased deviations are visible throughout the whole brain, with this low spatial frequency deviations being attributed to B1+ changes in the test retest condition. Although this particularly large residual bias field has been corrected in T1w/T2w BC, resulting in deviation maps with a very similar distribution to that seen in R1. The largest variances in Fig 5B and 5C were found in regions of high curvature and reduced cortical thickness (where the FreeSurfer segmentation variation can have an increased impact) and increased expected “myelination” values. The similarity between deviation maps can also be judged by the distributions of the deviation values. When evaluating the 25/50/75/98 percentiles of each distribution, 1.04/1.36/1.77/3.78% and 1.4/1.89/2.4/3.86% are found on the BC and non-BC T1w/T2 while 0.62/0.81/1.06/2.47% for the R1 maps. All these analyses suggest R1 to be the more reproducible myelin mapping approach.

### Left- Right hemisphere correlation

**Fig 6:**
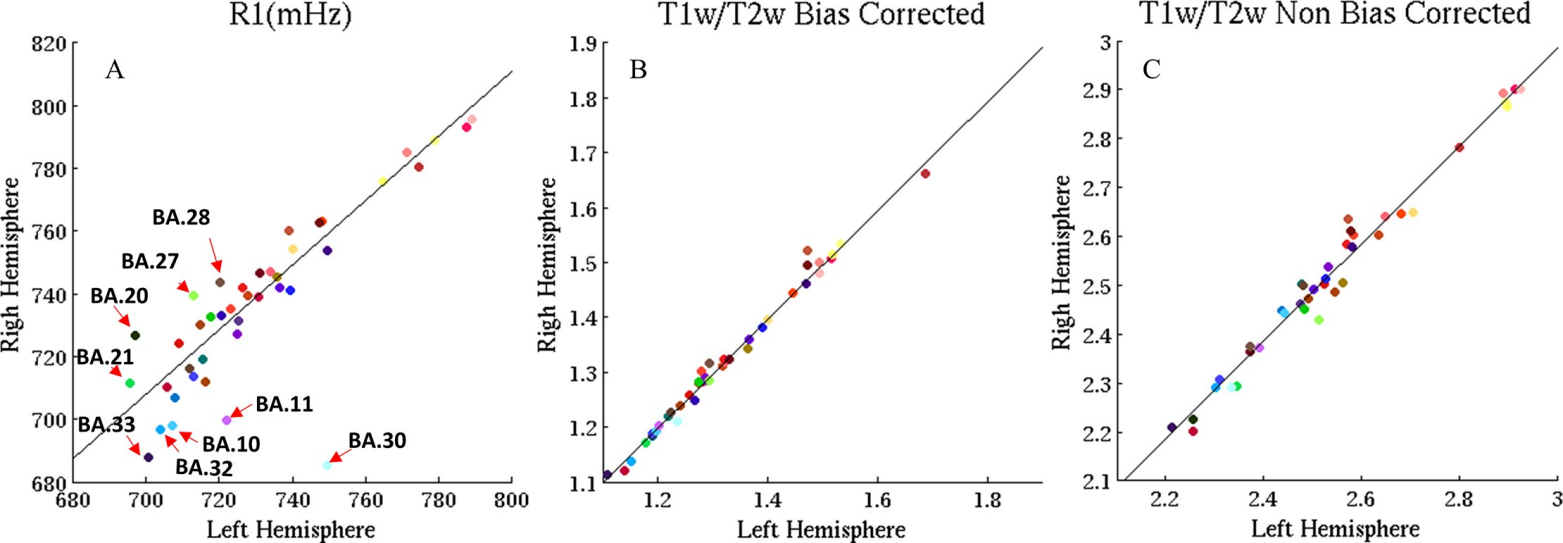
Left and right hemispheres relationship modelled by linear regression approach. (A) R1 (B) T1w/T2w with residual bias field correction and (C) original T1w/T2w maps. Some Brodmann regions with much deviation from the fitted curve in R1 are pointed by red arrows, mainly located in temporal and frontal lobes.

The cortical maps of the left and right hemispheres were compared to evaluate the intra-subject and intra-session robustness of the maps as well as a mean to evaluate the robustness of the obtained results to B1 inhomogeneities. The correlation between the left and right hemisphere for both methods is shown in Fig 6A–6C. The plots in Fig 6 contain the scatter points corresponding to the average value of vertices in Brodmann regions for R1 (A), bias corrected T1w/T2w (B) and original T1w/T2w (C) maps. These values are highly correlated between the left and right hemisphere in bias corrected T1w/T2w map (R^2^ ≈ 0.98). The effect of residual bias field on T1w/T2w map is a reduction in R^2^ (0.97). R1 maps have a lower inter hemispheric correlation (R^2^ ≈ 0.71) especially in regions such as BA10 (orbito-prefrontal region), 11 (orbitofrontal cortex), 20 (inferior temporal area), 21 (middle temporal area), 27 and 28, 30, 32 and 33 which lie mostly in frontal and temporal lobes (red arrows in Fig 6A). These are the regions of large B0 field inhomogeneity rather than regions of strong B1 field inhomogeneities (see S5 Fig). Large B0 inhomogeneities can impact the R1 mapping only by reducing the inversion efficiency of the inversion pulse (resulting in increased R1 values), while the effect on the signal intensity be cancelled in the division process of two images with the same echo time. The T1w/T2w maps are affected both due to the adiabatic pulse inversion efficiency and due to the T1w image being a gradient echo readout (susceptible to signal dephasing) while the T2w image is a spin echo sequence. Also, there was a clear tendency for increased R1 values in the right hemisphere with respect to the left hemisphere (see Fig 6A) where most points would be positioned above the identity line. An analysis performed on the correlation of the left vs right asymmetry of the various cortical maps with the asymmetry of B1 maps, showed that non bias-corrected T1w/T2w had the highest correlation (0.34), followed by R1 (0.13) and Bias Corrected T1w/T2w maps (0.03). This suggests that most of the asymmetry seen in Fig 6A is not explained by B1.

## Discussion

In this manuscript we have thoroughly compared two of the most commonly used protocols to perform cortical mapping. We have tried to address various aspects of their performance: the robustness to artifacts; reproducibility between and within subjects; ability to distinguish neighbouring cortical regions; the correlation between the two methods as well as the left vs right hemisphere symmetry. It should be noted that many of these findings, might only be valid at 3T and with the specific imaging protocols used, including also the calibration B_1_ and B_0_ maps.

To try to keep the comparison as focused on the contrast properties of the two approaches of cortical mapping, we have used a processing pipeline as close as possible to that provided by the HCP pipeline and have based all the R1 map analysis on the segmentation done on the T1w data. This choice was aimed at reducing variability associated with varying slice thicknesses attributed by freesurfer depending on the input image contrast [41] and the awareness that myelination values vary as a function of layer depth [6,26,42,43]. These analyses were done considering a Brodmann atlas parcellation of the brain that might not be an ideal parcellation, because myelin distribution is known not to follow exactly the cytoarchitecture and, more importantly, this atlas was mapped onto the cortical surface by David Van Essen in the late 1990s as an educated guess of the cytoarchitecture parcellation performed by Brodmann in the 1900 without the advanced neuroimaging methods available today. On the other hand, using this atlas rather than the HCP multi-model parcellation atlas [44] that was obtained using one of the methods being tested in this comparison, avoids potential biases in the comparison between the two cortical myelin mapping methods. One unfortunate consequence of our choice is, for example, that while area 3a appears more distinct from area 3b in T1w/T2w vs R1, this will appear as noise on the statistical tests involving Brodmann’s area 3 presented in Table 2.

To make the observations using the two methodologies and metrics comparable, we have used the slope calculated in Eq 2 and Eq 3 as a conversion factor. We observed that the standard deviation within each Brodmann region was comparable for both methods (see Fig 4A and 4C), which was the case for variance over the population (Fig 4D), but the bias corrected T1w/T2w approach presented a lower standard deviation between subjects (by a factor of ~0.38) than the MP2RAGE longitudinal relaxation rate method. When looking at the reproducibility over the same subjects (see Fig 5), test-retest, as computed by the deviation maps, it can be seen that the increase in variability introduced by the T1w/T2w protocol was considerable when the remaining bias field was not corrected.

Our analysis on the impact of motion restriction show that motion restriction reduced the number of datasets that had to be discarded and that an increased number of datasets using the longer MP2RAGE sequence had to be discarded. This suggests that when using protocols that are based on one single (longer) acquisition, it is beneficial to use motion tracking, be it camera based or navigator based to reduce the presence of such artifacts [45,46]. Such methods are routinely used now both at the various research sites throughout the world (including the HCP initiative) and show benefits even for shorter acquisitions, but here we have limited our comparison to sequences distributed as part of the manufacturer product. Yet, surprisingly, the presence or absence of such artifacts did not have a significant impact neither on the average value nor on the standard deviations of each Brodmann regions. This can be observed by both the good correlation between the test and retest measurements and standard deviations both when using the full 17 subjects (Fig 3) or when analysing simply the artifact-free datasets (S4 Fig).

For the first time, we have looked at the correlation and conversion factors between cortical maps obtained with T1w/T2w maps and R1 maps. Both a visual and analytical strong correlation between the two metrics was found, with the regions having the biggest deviations being those associated with increased transmit B1 field. Because the transmit B1 field is explicitly taken into account when calculating the R1 mapping (with only the impact on the adiabatic pulse being ignored), it would be expected that the major bias resides on the T1w/T2w maps.

The left right hemisphere asymmetry in cortical values observed at 3T has also been observed at 7T using MP2RAGE [26] and reported by various other studies where R1 of the right hemisphere was greater than of the left hemisphere [47–49]. It should be noted that the studies from Garber et al. and Kim et al. used saturation recovery and inversion recovery to measure T1 [48,49]. Interestingly, this difference is not observed when using the T1/T2w maps, despite these maps containing a very similar type of T1w contrast (obtained with an MPRAGE image) as that present on the MP2RAGE R1 maps. Other than this systematic difference between hemispheres observed in the R1 maps, the remaining differences (BA10, 11, 32 and 33) were found in regions mostly associated with large B0 inhomogeneities (see S5 Fig). This can be attributed to the B0 sensitivity of the particular parameter set chosen for the MP2RAGE R1 maps, which is computed by combining two images with echo times of 3.3ms while the T1w image was calculated with a 2.6ms echo time and the T2w turbo spin echo sequence is B0 field insensitive.

In this manuscript, we used FreeSurfer folding-based surface registration to the fsaverage surface template. Although this surface-based approach has the benefit of improved alignment of cortical shape in comparison with volumetric methods, it is not a perfect technique for generating average myelin maps due to inconsistency in the number of anatomical folds across subjects in several cortical areas such as hOC5 region, Brodmann areas 44 or 45 and MT +/V5 on the lateral occipital cortex which is characterized by high inter-subject variability in cortical folding. MSM-sulc or other Multimodal surface matching (MSM) method (e.g. MSM-myelin to align cortical regions such as MT+ with poor overlap across subjects) would be preferable for better cross-subject alignments [50].

The general findings of this paper at 3T (and with these particular sequence parameters) are not straightforward to extrapolate to higher fields or even to sequence protocols with other parameters. As the field increases, so will the B1 and B0 inhomogeneities. Thus, transmit field B1 biases will tend to increase further in the T1w/T2w approach where the transmit field bias is not explicitly corrected (at 3T it was responsible for some of the differences found in respect to the R1 maps). On the other hand, the increase in B0 inhomogeneities will probably affect the R1 based MP2RAGE more (as suggested in some of the left right asymmetries observed), although this could be minimised by using protocols with shorter echo times.

## Conclusion

Both cortical mapping methods show similar and highly replicable cortical maps enhancing regions traditionally found as being highly myelinated. T1w/T2w maps were shown to be more robust to motion related artifacts cohort, which was attributed to relying on two separate acquisitions. Removing the residual bias field from T1w/T2w maps is an essential step for within subject reproducibility and reliable cortical segmentation, at the cost of removing natural variations that exist between subjects. R1 maps showed more reproducibility in test re-test experiments within the same subject. The ability to differentiate across neighbouring cortical regions was found to be comparable with R1 maps having stronger differentiation in occipital areas and T1w/T2w being superior in the central sulcus. This could make R1 a preferable metric in longitudinal studies while T1w/T2w maps (bias corrected) showed a higher reproducibility of the average myelination across subjects for different Brodmann areas which could make it a preferable metric for cortical segmentation.

## Supporting information

S1 Fig Human cortical parcellations. (A) VDG11b 52-surface-mapped cortical areas. (B) Brodmann (1909) areas.
(TIF)

S2 Fig R1 and T1w/T2w Cortical maps in medial view (left hemisphere). Medial view of R1, T1w/T2w (with and without residual bias field correction) cortical maps (shown in columns) generated for 5 individual subjects (shown in rows) represented in the high resolution (~164k) fs_LR inflated surface. The last two rows show the average maps and their cortical surface gradient.
(TIF)

S3 Fig R1 and T1w/T2w Cortical maps for right hemisphere. Lateral (A) and Medial (B) view of right hemisphere for R1, T1w/T2w (BC and non-BC) cortical maps (shown in columns) generated for 5 individual subjects (shown in rows) represented in the high resolution (~164k) fs_LR inflated surface.
(TIF)

S4 Fig Test (Fixation) vs. Retest (Non-Fix) for artefact-free scans. (A, D) R1 map. (C, F) original T1w/T2w cortical map. (B, E) Bias corrected T1w/T2w map. (A-C) standard deviation in each Brodmann region on group average multiplied by 0.3. (D-F) Deviation from the mean value of vertices in each cortical area across the subjects multiplied by 0.3.
(TIF)

S5 Fig **Surface demonstration of B0 field map (A) and B1 transmit field (B) in lateral (top rows) and medial views (bottom rows).** (A) B0 field map of the left and right hemisphere (left and right columns) with high signal intensity in frontal lobe. (B) B1 map with intensity variations in temporal and cingulate region. Both maps are the average maps across all subjects displayed on the 164k-fs_LR inflated surface.
(TIF)
