# Supplementary figures and images for: A comparison of in vivo MRI based cortical myelin mapping using T1w/T2w and R1 mapping at 3T
Source: PLoS One. 2019 Jul 3;14(7):e0218089. doi: 10.1371/journal.pone.0218089 (PMC6609014; doi:10.1371/journal.pone.0218089)

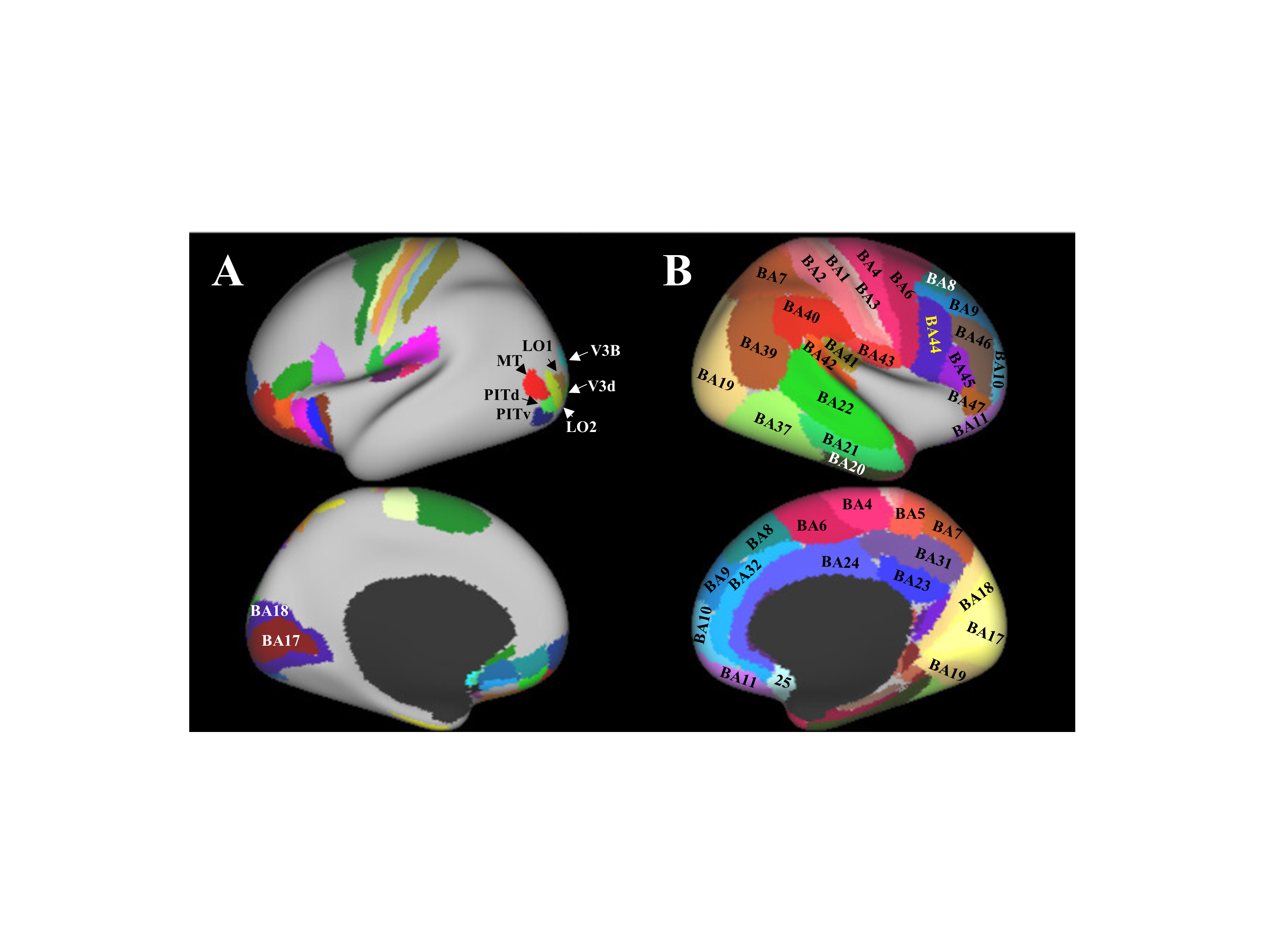

Supplement: S1 Fig — (A) VDG11b 52-surface-mapped cortical areas. (B) Brodmann (1909) areas. (TIF) [file pone.0218089.s001.tif]

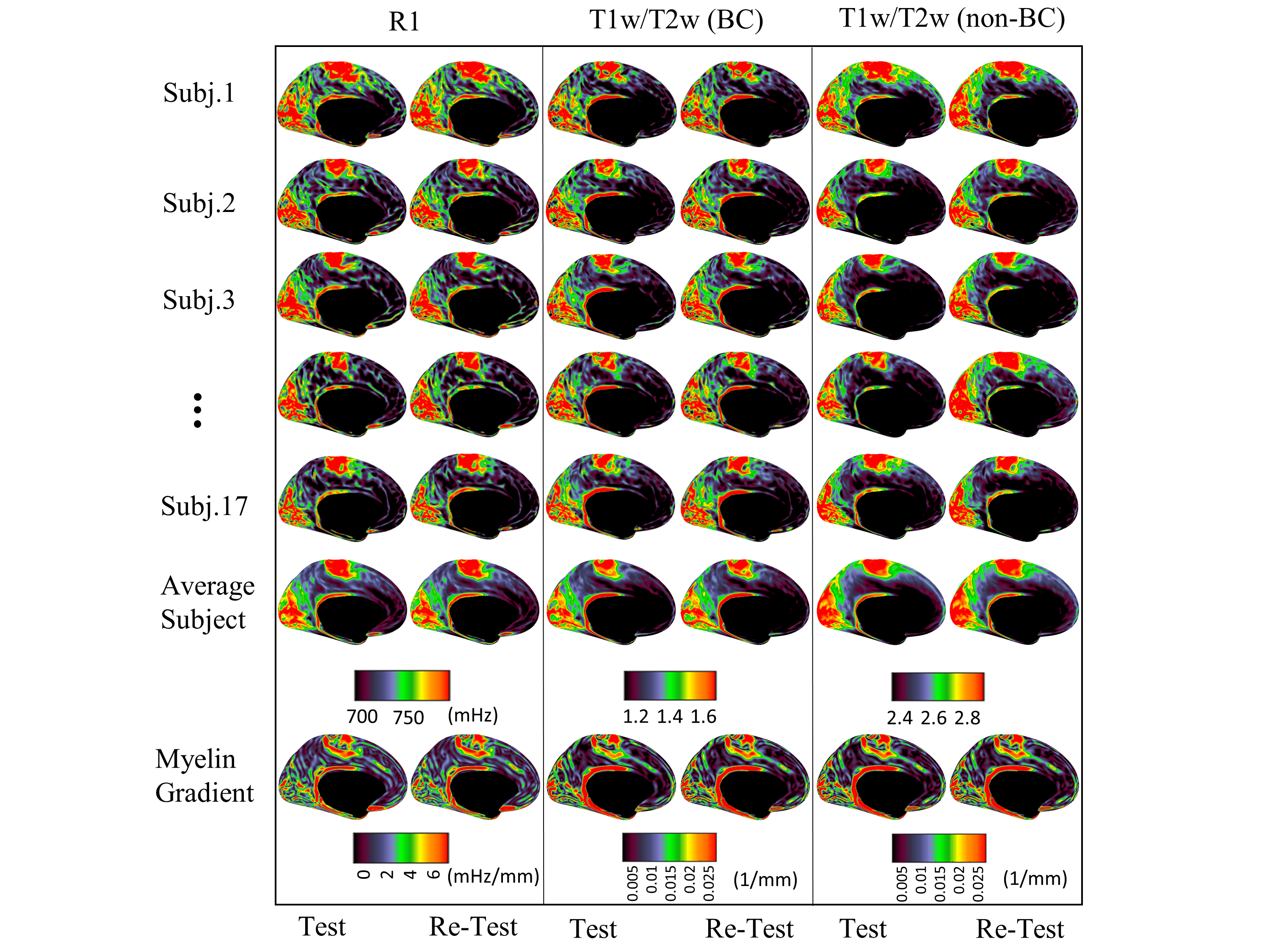

Supplement: S2 Fig — Medial view of R1, T1w/T2w (with and without residual bias field correction) cortical maps (shown in columns) generated for 5 individual subjects (shown in rows) represented in the high resolution (~164k) fs_LR inflated surface. The last two rows show the average maps and their cortical surface gradient. (TIF) [file pone.0218089.s002.tif]

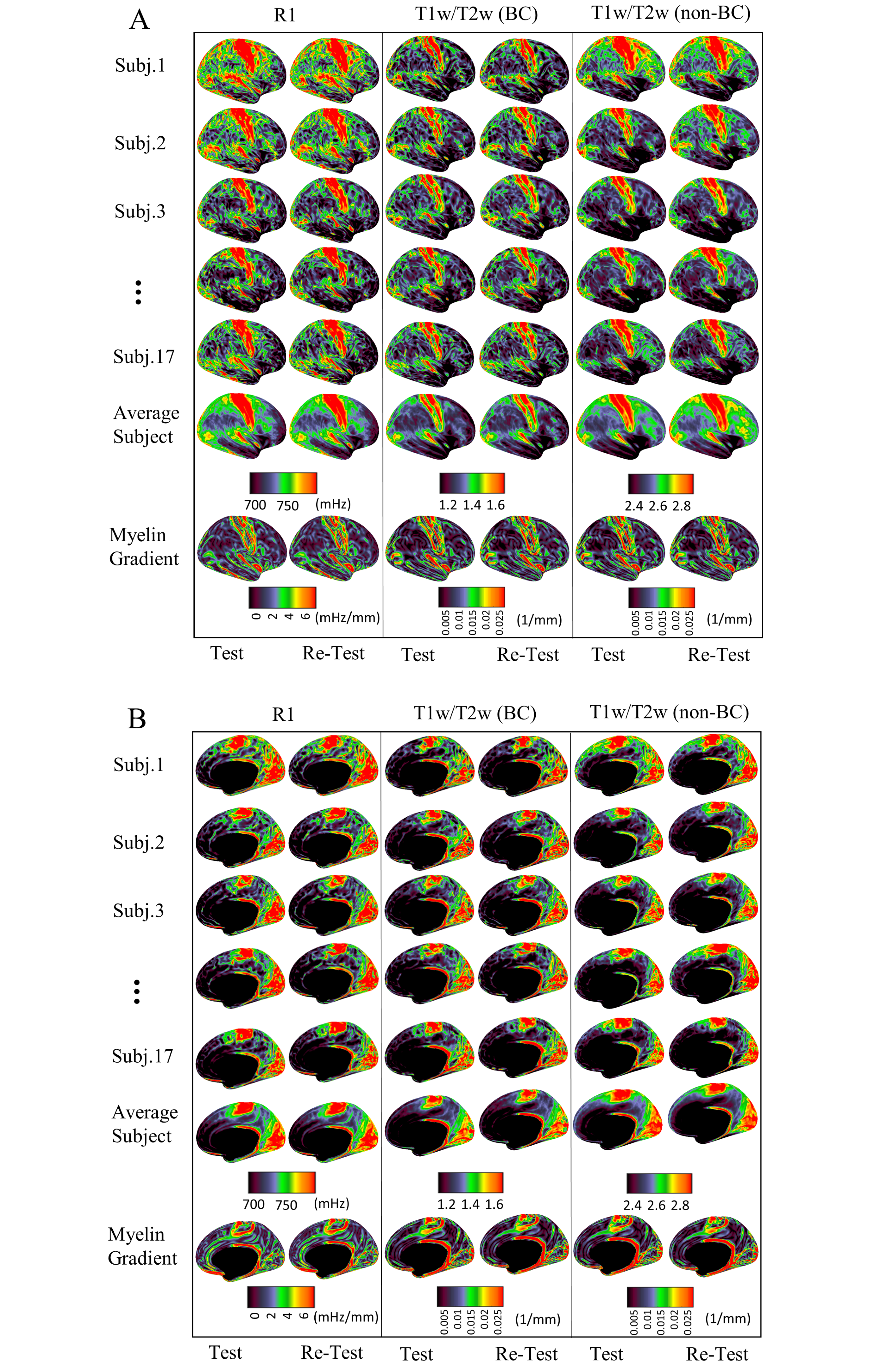

Supplement: S3 Fig — Lateral (A) and Medial (B) view of right hemisphere for R1, T1w/T2w (BC and non-BC) cortical maps (shown in columns) generated for 5 individual subjects (shown in rows) represented in the high resolution (~164k) fs_LR inflated surface. (TIF) [file pone.0218089.s003.tif]

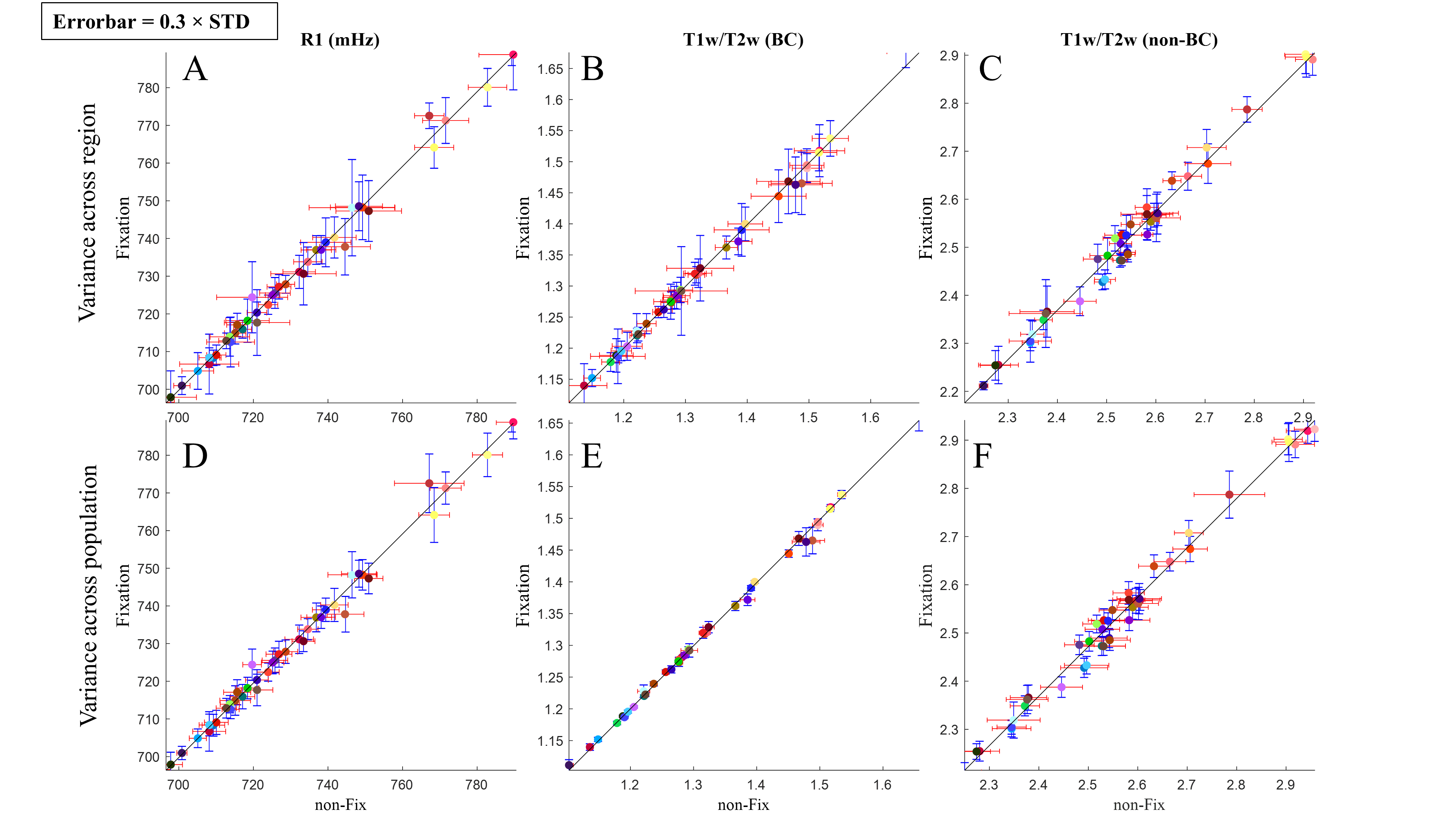

Supplement: S4 Fig — (A, D) R1 map. (C, F) original T1w/T2w cortical map. (B, E) Bias corrected T1w/T2w map. (A-C) standard deviation in each Brodmann region on group average multiplied by 0.3. (D-F) Deviation from the mean value of vertices in each cortical area across the subjects multiplied by 0.3. (TIF) [file pone.0218089.s004.tif]

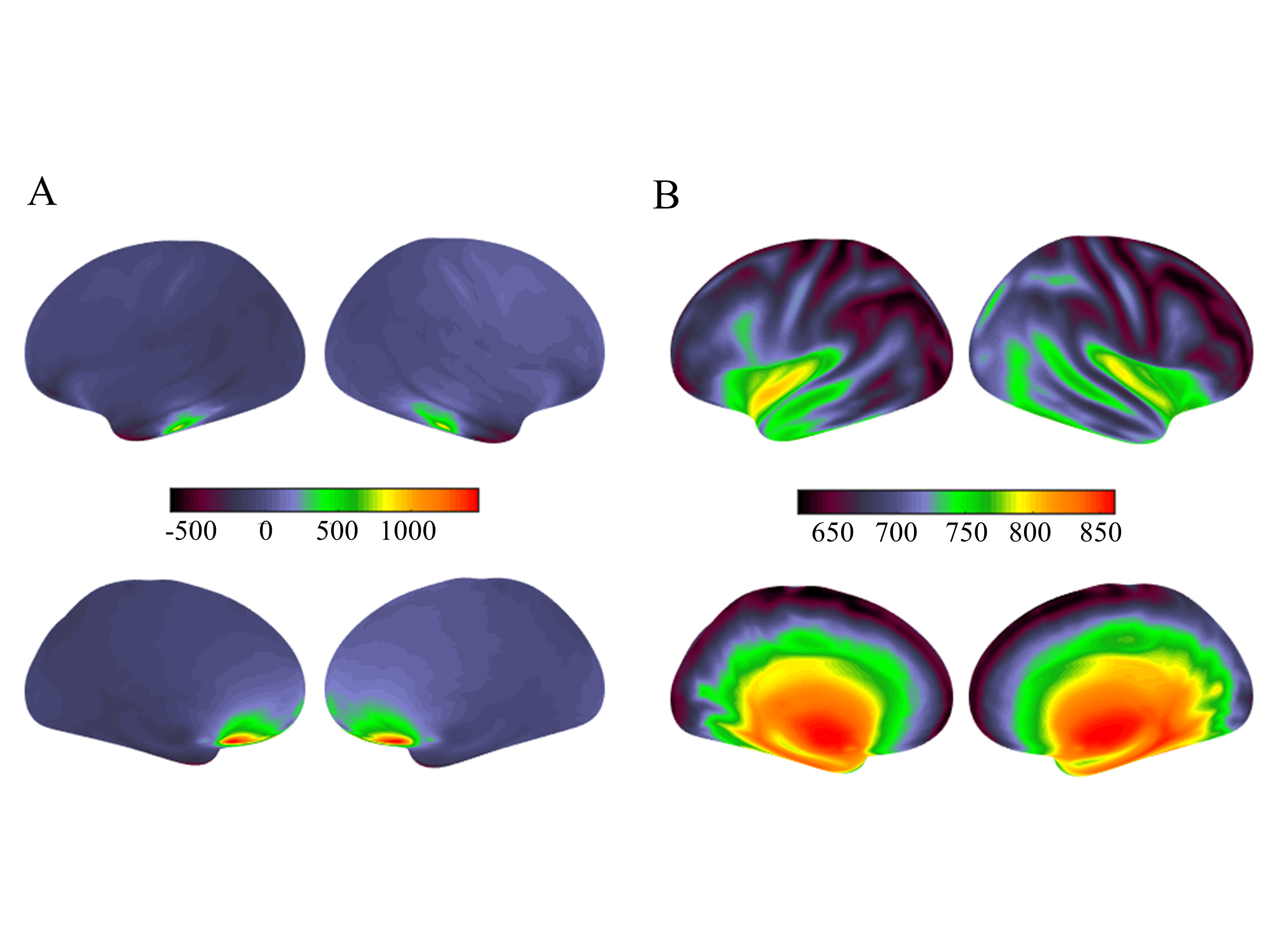

Supplement: S5 Fig — Surface demonstration of B0 field map (A) and B1 transmit field (B) in lateral (top rows) and medial views (bottom rows). (A) B0 field map of the left and right hemisphere (left and right columns) with high signal intensity in frontal lobe. (B) B1 map with intensity variations in temporal and cingulate region. Both maps are the average maps across all subjects displayed on the 164k-fs_LR inflated surface. (TIF) [file pone.0218089.s005.tif]
